# Primary Dedifferentiated Liposarcoma of the Posterior Mediastinum With a Positive Surgical Margin

**DOI:** 10.7759/cureus.36611

**Published:** 2023-03-23

**Authors:** Ryusei Yoshino, Nana Yoshida, Shunsuke Yasuda, Akane Ito, Masaki Nakatsubo, Masahiro Kitada

**Affiliations:** 1 Thoracic Surgery and Breast Surgery, Asahikawa Medical University Hospital, Asahikawa, JPN

**Keywords:** radiotherapy, positive surgical margin, posterior mediastinum, dedifferentiated liposarcoma, liposarcoma

## Abstract

Liposarcoma often occurs in the extremities and retroperitoneum. Primary mediastinal liposarcoma is uncommon, and there is no settled opinion regarding adjuvant therapy after surgery. We have recently experienced a relatively rare case of primary dedifferentiated liposarcoma of the posterior mediastinum. The patient was a 76-year-old woman. An abnormal shadow was noted in the posterior mediastinum. Esophageal submucosal tumor and gastrointestinal stromal tumor were suspected; endoscopic ultrasound-guided fine needle aspiration was performed, but a definitive diagnosis could not be obtained. As the tumor tended to slowly grow, surgical resection was performed. Based on histopathological findings, the patient was diagnosed with primary dedifferentiated liposarcoma of the posterior mediastinum. Owing to the presence of a positive surgical margin, postoperative radiotherapy (60 Gy/24 fr/6 w) was administered. No recurrence was observed after three and a half years of follow-up. The prognosis of primary dedifferentiated liposarcoma of the posterior mediastinum with a positive surgical margin is poor, but postoperative radiotherapy may be useful.

## Introduction

Liposarcoma often occurs in the extremities and retroperitoneum. Primary mediastinal liposarcoma is a relatively rare type of disease and accounts for less than 1% of all types of mediastinal tumors [1]. The most common histological type is well-differentiated liposarcoma [2]. The present case was a relatively rare histological type of primary dedifferentiated liposarcoma of the posterior mediastinum. There is no consensus on the postoperative adjuvant therapy for primary dedifferentiated liposarcoma of the posterior mediastinum, and only a few studies have reported on this topic. Here, we report a case of a patient with primary dedifferentiated liposarcoma of the posterior mediastinum treated with postoperative radiotherapy along with a review of the relevant literature.

## Case presentation

**Figure 1:**
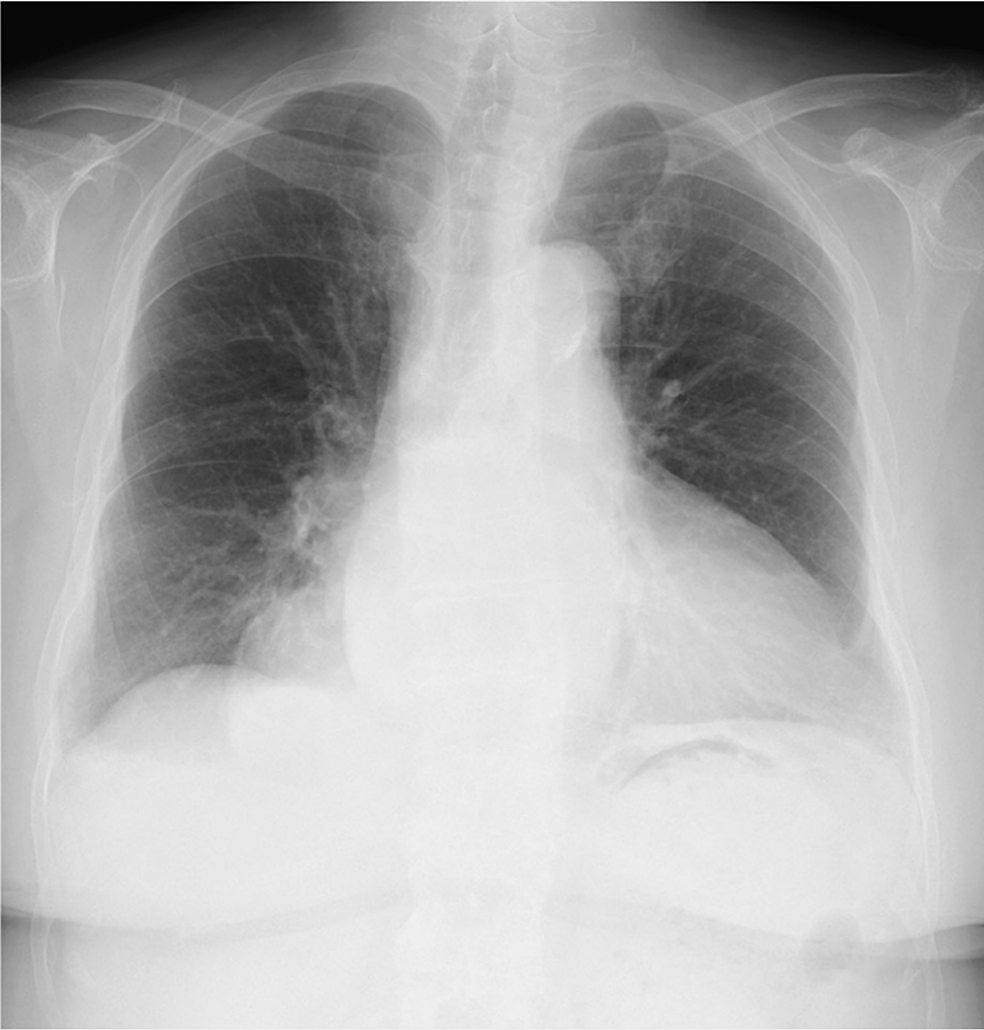
Chest radiograph (frontal view) An abnormal shadow is observed on the right lower lung field.

**Figure 2:**
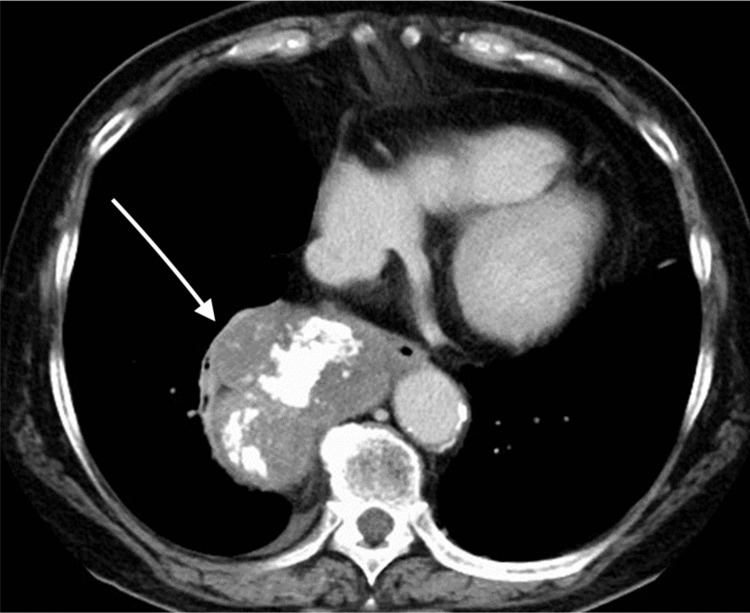
Chest computed tomography findings (mediastinal condition) A mass measuring 110 mm × 90 mm × 80 mm with internal calcification is observed on the right posterior mediastinum.

The patient was a 76-year-old woman. A chest X-ray during a health checkup revealed an abnormal shadow in the right lower lung field (Figure 1). A closer examination and chest computed tomography (CT) scan revealed a lesion measuring 110 mm × 90 mm × 80 mm with internal calcification in the right posterior mediastinum (Figure 2). Esophageal submucosal tumor and gastrointestinal stromal tumor (GIST) were suspected, and endoscopic ultrasound-guided fine needle aspiration (EUS-FNA) was performed; however, a definitive diagnosis could not be obtained. The tumor tended to grow slowly over a period of six months; hence, surgical resection was performed. The patient was previously diagnosed with hypertension, dyslipidemia, and benign goiter. Her family history was unremarkable. The patient had a smoking history of 20 cigarettes/day and had been a smoker for 56 years (Brinkman Index, 1120). Upon admission, her height was 155 cm, weight was 61.0 kg, and body mass index was 25.4. No difference was observed in the breath sounds auscultated between the right and left chest, and no abnormality was noted. Laboratory findings on admission showed normal blood count, biochemistry, or coagulation tests. The levels of tumor markers (carcinoembryonic antigen and cancer antigen 15-3) were not elevated. No abnormal findings were observed during the assessment of respiratory function or electrocardiographic examination.

**Figure 3:**
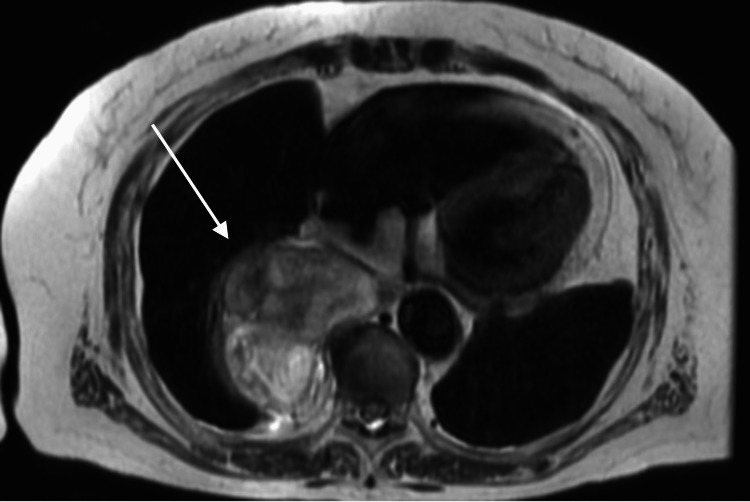
Chest magnetic resonance imaging (T2-weighted imaging) A lobulated mass with a well-defined margin is observed in the right posterior mediastinum, showing uneven mild hyperintensity. The lesion is adjacent to the esophagus, and findings suggestive of a beak sign are observed.

**Figure 4:**
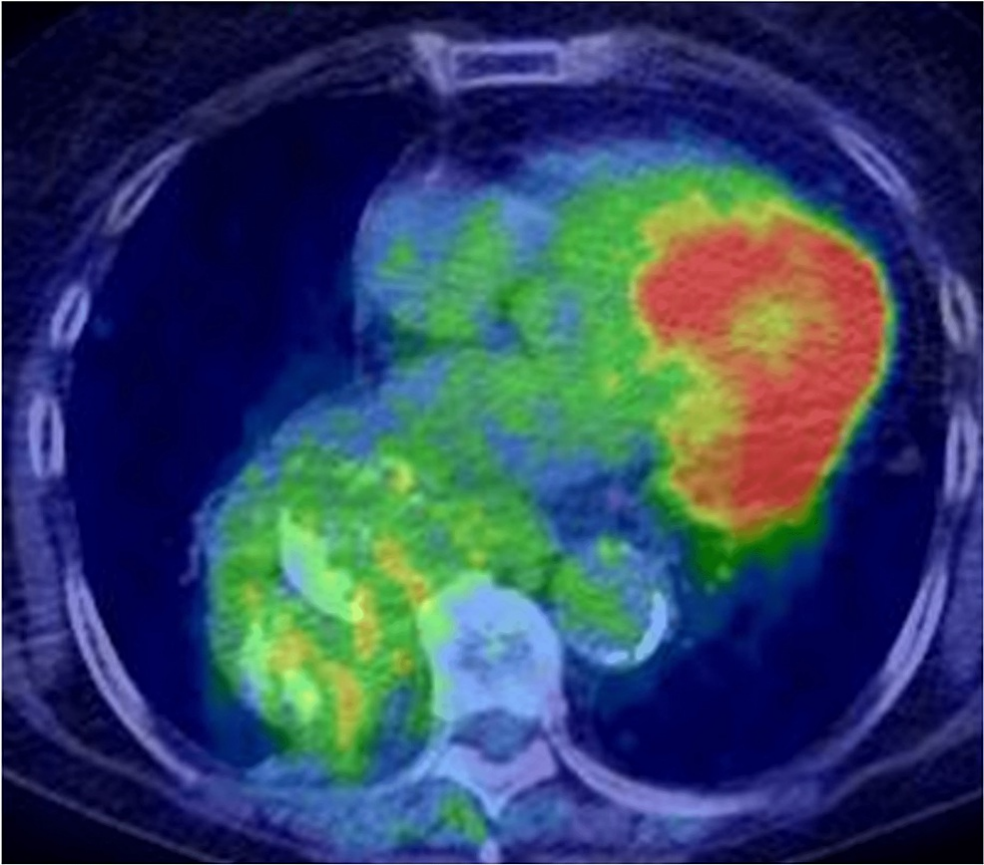
Fluorodeoxyglucose-positron emission tomography findings Accumulation of fluorodeoxyglucose with a Max SUV of 3.4 is observed in the right posterior mediastinum. Max SUV: maximum standardized uptake value

Chest magnetic resonance imaging (MRI) revealed a lobulated mass with a clear margin in the right posterior mediastinum. A heterogeneous mild hyperintensity was observed on T2-weighted imaging (Figure 3) and a mild hypointensity on T1-weighted imaging. The lesion was adjacent to the esophagus. Fluorodeoxyglucose-positron emission tomography showed the accumulation of fluorodeoxyglucose with the maximum standardized uptake value of 3.4 in the right posterior mediastinum (Figure 4). Upper gastrointestinal tract endoscopy showed no obvious mucosal lesion. No malignant findings were observed on EUS-FNA. Since the tumor margins were clear on the thoracic MRI and there was no evidence of invasion, complete resection was considered feasible. Based on the above results, a definitive preoperative diagnosis was not obtained; however, posterior mediastinal tumor resection was performed considering the possibility of esophageal GIST.

**Figure 5:**
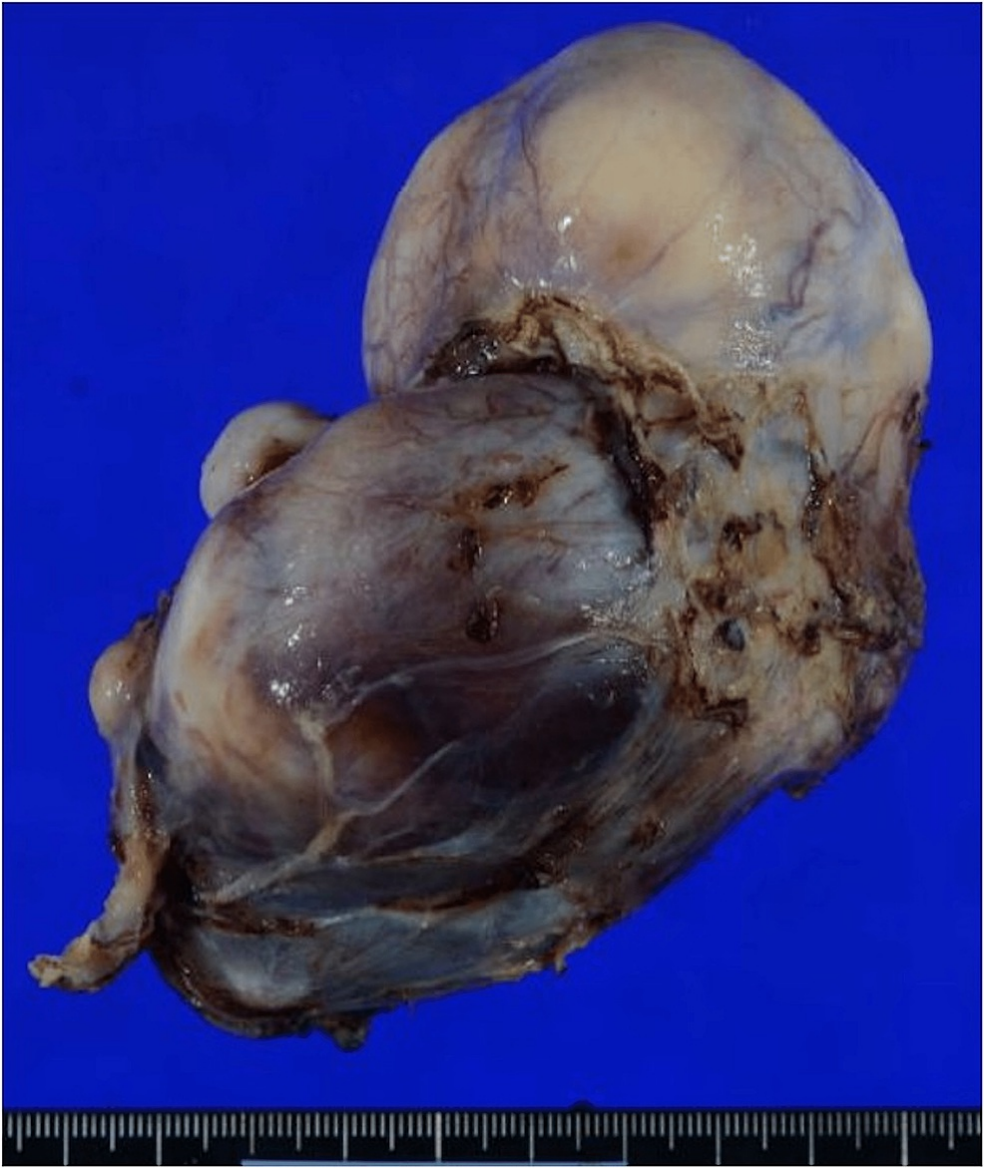
Findings of tumor resection An encapsulated yellow to grayish white lobulated mass with internal calcification and ossification, measuring 11 cm × 7 cm× 4 cm, is observed.

In accordance with the intraoperative findings, thoracotomy was performed by creating a 13-cm incision at the fifth intercostal space with the thoracoscope placed at the left lateral position. The tumor adhered to the right lower lobe, posterior mediastinal pleura, and right lower pulmonary vein. The adhesions were dissected circumferentially. Since the adhesion between the tumor and the right lower lobe was relatively strong and infiltration was suspected, partial combined resection was performed. By contrast, the esophagus and carina, which were located at the deepest part, were easily dissected, and the tumor was finally resected. The adhesiolysis site of the esophagus was covered with pericardial fat pad. The surgery lasted for one hour and 53 minutes, and the amount of blood loss was 20 mL. The resected specimen appeared as an encapsulated yellow-to-grayish white lobulated mass, with calcification and ossification, and measured 11 cm × 7 cm × 4 cm (Figure 5). 

**Figure 6:**
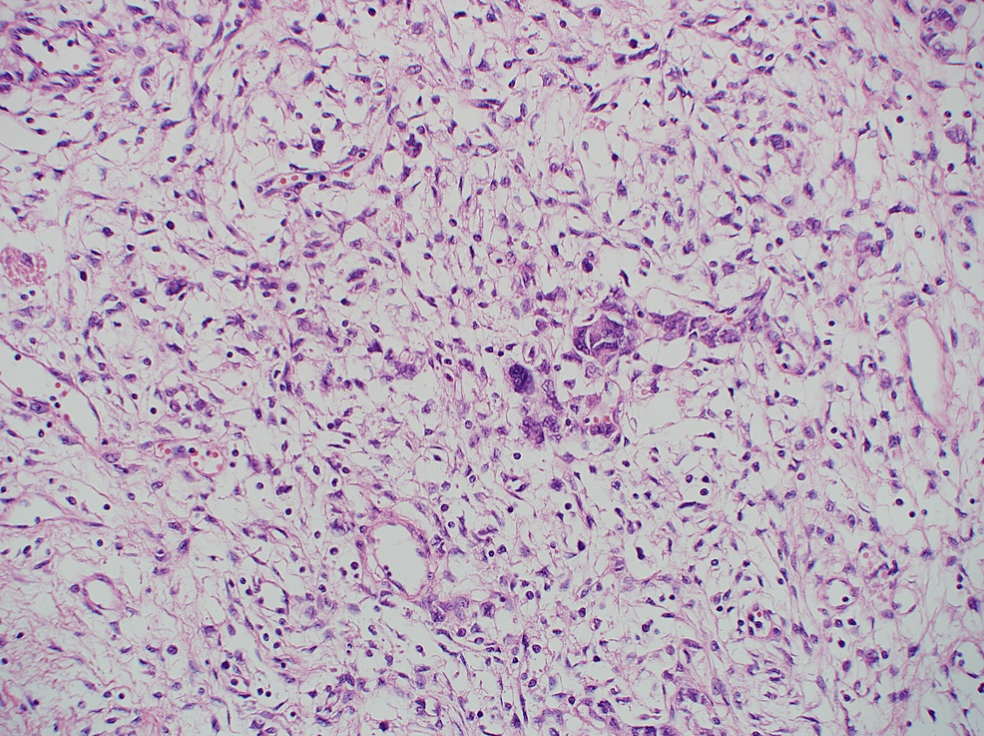
Histopathological findings The spindle cells proliferated in a complicated fascicular manner with condensation and rarefaction (hematoxylin and eosin staining, ×20).

**Figure 7:**
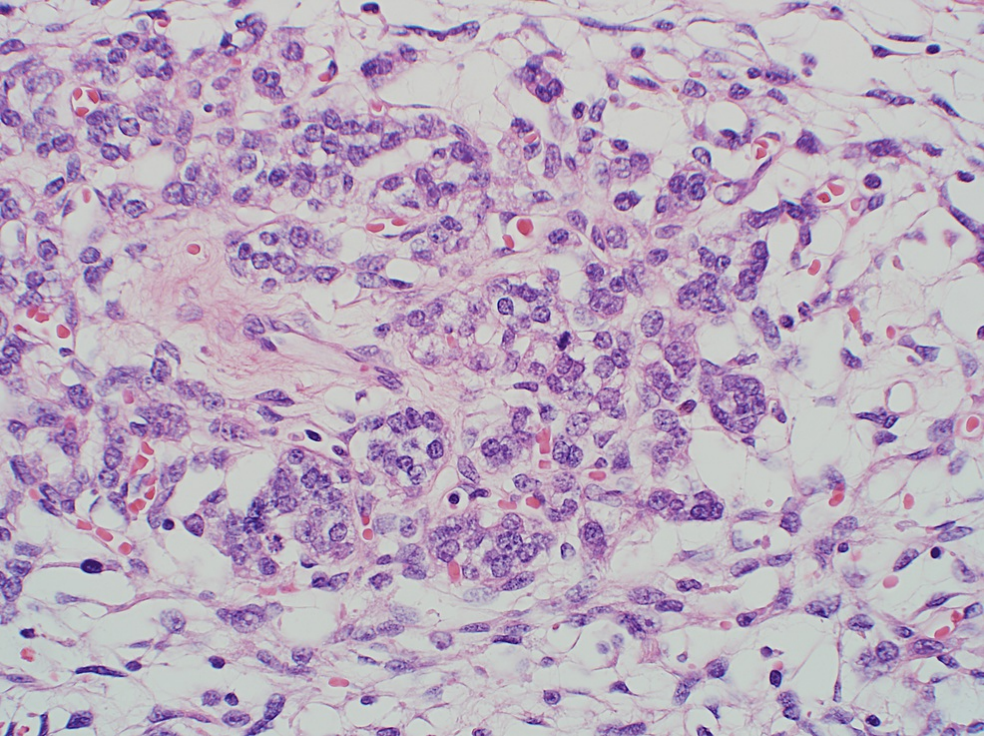
Histopathological findings Seven mitotic figures in 10 high-power fields are observed (hematoxylin and eosin staining, ×40).

**Figure 8:**
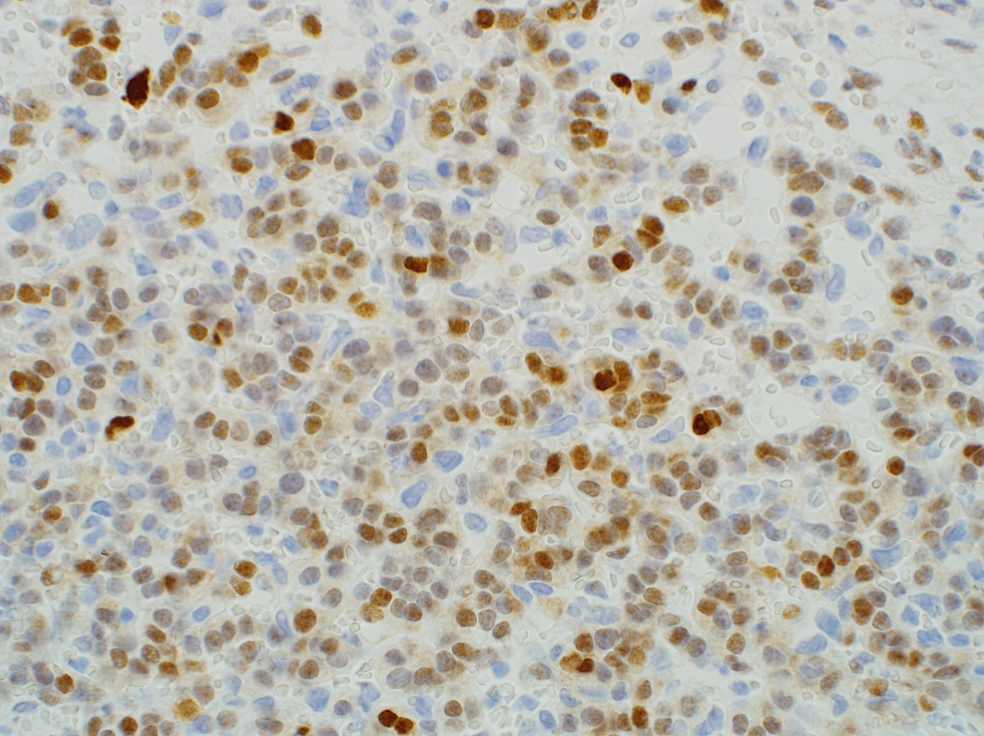
Histopathological findings Mouse double minute 2 (MDM2) is highly expressed (×40).

**Figure 9:**
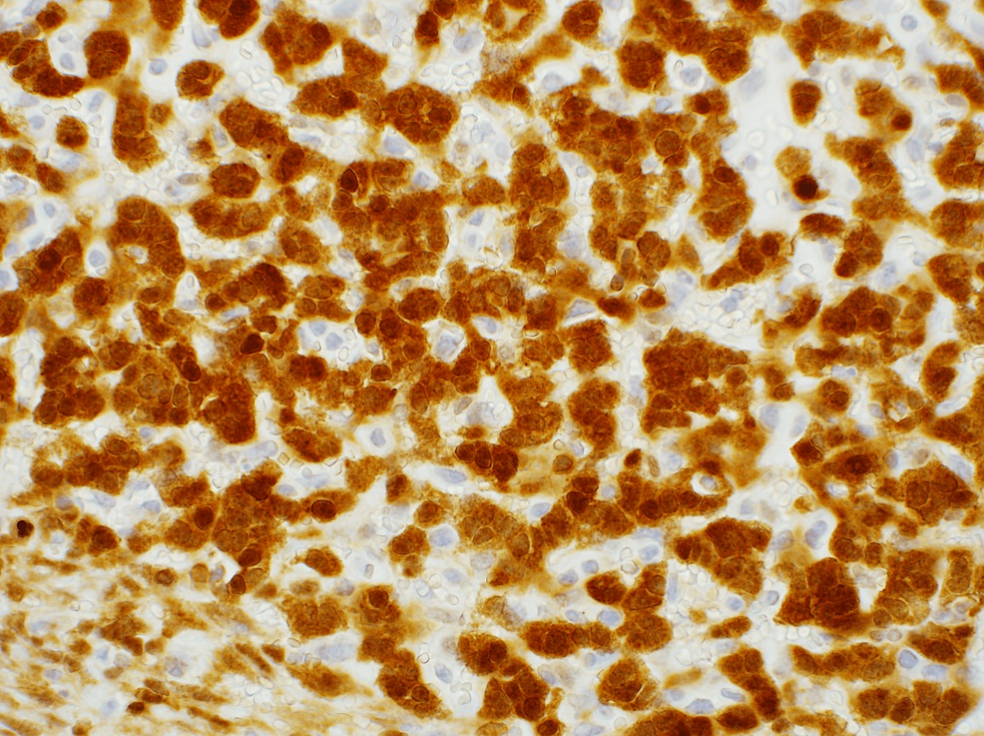
Histopathological findings Cyclin-dependent kinase 4 (CDK4) is highly expressed (×40).

Histopathological examination showed fibrous and adipose tissues in the surface layer of the tumor and tumor growth in the center. Hematoxylin and eosin staining of the tumor cells showed proliferation of spindle cells arranged in a complicated fascicular manner, with condensation and rarefaction. The tumor showed extensive irregular bone differentiation and slight necrotic foci. Myxoid change was observed in some areas of the fatty tissue in the tumor margins, and atypical cells with spindle nuclei were sporadically observed in the fibrous septa. Seven mitoses in 10 high-power fields were noted. The tumor had indistinct borders, and partially positive margins were observed on the caudal side of the tumor. Immunohistochemistry revealed spindle-shaped atypical cells that were strongly positive for mouse double minute 2 (MDM2) and cyclin-dependent kinase 4 (CDK4). However, the patient tested negative for h-caldesmon, desmin, cluster of differentiation 34 (CD34), S-100, and signal transducer and activator of transcription 6 (Figures 6-9).

The chest tube was removed on postoperative day two, and the patient was eventually discharged on postoperative day eight. After surgery, radiotherapy (60 Gy/24 fr/6 w) was performed by a respiratory physician. Since the patient was undergoing radiation therapy for a posterior mediastinal tumor, periodic echocardiography was performed to monitor for cardiotoxicity. No decline in cardiac function was observed after the completion of radiotherapy. Every six months, the patient underwent a chest CT as a routine examination. No recurrence was observed after three and a half years of follow-up.

## Discussion

The incidence of soft tissue sarcomas (STS) is approximately 40 cases per one million population per year, accounting for approximately 1.5% of all types of malignant tumors [3]. The common types of STS are undifferentiated pleomorphic sarcoma (24%), liposarcoma (14%), and leiomyosarcoma (8%), in descending order of frequency [4]. Liposarcoma often occurs in the extremities and retroperitoneum. Primary mediastinal liposarcoma is a rare type of disease and accounts for less than 1% of all types of mediastinal tumors [1].

Primary liposarcoma of the mediastinum is commonly found after tumor growth due to the occurrence of symptoms such as dyspnea, chest pain, and tachycardia caused by compression and infiltration of the intrathoracic structures [5,6]. Chest CT is a useful method for preoperative diagnosis. In patients with liposarcoma, the CT values are close to those of adipose tissues, and the extent of tumor growth and presence/absence of invasion to adjacent organs can be evaluated by plain CT. Chest MRI is useful for determining the degree and extent of infiltration into the adjacent large blood vessels and luminal organs. However, differentiation from other soft tissue tumors is difficult, as the images obtained by chest MRI may not provide additional qualitative data [7]. In our present case, chest CT showed a calcified mass, while chest MRI showed a beak-like narrowing of the lower esophagus in close proximity to the posterior mediastinal tumor. Thus, esophageal GIST was considered as a differential diagnosis. Esophageal resection was also considered at the time of surgery.

Liposarcoma is mainly classified into five types: well-differentiated, myxoid, round cell, pleomorphic, and differentiated. Immune markers for specific tumor types, such as MDM2 and CDK4 for dedifferentiated liposarcoma, are often used for diagnosis [8]. Ortega et al. reported that well-differentiated liposarcoma was the most common histological type, accounting for 55% of primary tumors of the posterior mediastinum [2]. In terms of local recurrence and distant metastasis, the dedifferentiated type tends to have a poorer prognosis than the well-differentiated type [9].

In this patient, a posterior mediastinal tumor was incidentally found in the absence of any symptoms. Through the conduct of imaging examinations, esophageal GIST was listed as a differential diagnosis, but a definitive diagnosis was not made. However, surgical resection was still performed. This was a relatively rare case of primary dedifferentiated liposarcoma of the posterior mediastinum, a tumor with a relatively poor prognosis, diagnosed based on the postoperative histopathological findings.

The first-line treatment for primary liposarcoma of the posterior mediastinum is complete surgical resection [10]. Several studies reported that surgical complete resection is the only radical treatment for the disease [11]. The efficacy of postoperative adjuvant therapy in liposarcoma has not been established because both radiotherapy and chemotherapy are less sensitive to the disease. However, other previous studies reported the administration of postoperative radiotherapy after surgical resection and radiotherapy for postoperative recurrence [11,12].

Ortega et al. reported that 11 of 18 patients with primary liposarcoma of the posterior mediastinum underwent complete resection, but only four of them had negative surgical margins [2]. This may be because the posterior mediastinum does not allow sufficient visualization of the surgical field, and it is difficult to secure a space for operative manipulation, which increases the possibility of residual tumors.

Because the surgical margin was positive, postoperative radiotherapy (60 Gy/24 fr/6 w) was administered in this patient, owing to the increased risk of recurrence. Only a few patients received radiotherapy after surgery [11]. The patient in this case report survived without recurrence after three and a half years of follow-up. Radiotherapy for mediastinal tumors may affect the adjacent organs, such as the lungs, esophagus, and heart, with high radiosensitivity; therefore, caution is required when administering this treatment. This case may indicate the effectiveness of postoperative radiotherapy in a patient with a positive surgical margin. Therefore, a continuous follow-up is recommended by performing periodic CT examinations, etc., every six months.

## Conclusions

This was a case of a patient with primary dedifferentiated liposarcoma of the posterior mediastinum, a tumor with a poor prognosis, in whom posterior mediastinal tumor was incidentally found in the absence of any symptoms. Our findings suggest that postoperative radiotherapy may be effective in patients with positive surgical margins. Further studies are warranted to accumulate relevant data related to this case in order to generalize the use of adjuvant therapy in patients with primary dedifferentiated liposarcoma of the posterior mediastinum.

## References

[1] Barbetakis N, Samanidis G, Paliouras D, Boukovinas I, Kiziridou A, Tsilikas C (2008). A rare cause of mediastinal mass: primary liposarcoma. J BUON.

[2] Ortega P, Suster D, Falconieri G, Zambrano E, Moran CA, Morrison C, Suster S (2015). Liposarcomas of the posterior mediastinum: clinicopathologic study of 18 cases. Mod Pathol.

[3] Ferrari A, Sultan I, Huang TT (2011). Soft tissue sarcoma across the age spectrum: a population-based study from the Surveillance Epidemiology and End Results database. Pediatr Blood Cancer.

[4] Kransdorf MJ (1995). Malignant soft-tissue tumors in a large referral population: distribution of diagnoses by age, sex, and location. AJR Am J Roentgenol.

[5] Chaput-Dugas ME, Chughtai T, Liberman M, Duranceau A, Martin J, Barkat F, Ferraro P (2010). Successful resection of a large incidentally found primary mediastinal liposarcoma. J Surg Case Rep.

[6] Keita P, Tran A, Cheema M, Peterman NJ, Katigbak M (2022). Mediastinal liposarcoma with anterior and posterior mediastinal involvement: a thoracic oncovascular case report. Cureus.

[7] Burkill GJ, Badran M, Al-Muderis O, Meirion Thomas J, Judson IR, Fisher C, Moskovic EC (2003). Malignant gastrointestinal stromal tumor: distribution, imaging features, and pattern of metastatic spread. Radiology.

[8] Hornick JL (2014). Novel uses of immunohistochemistry in the diagnosis and classification of soft tissue tumors. Mod Pathol.

[9] Dei Tos AP (2000). Liposarcoma: new entities and evolving concepts. Ann Diagn Pathol.

[10] Chiyo M, Fujisawa T, Yasukawa T (2001). Successful resection of a primary liposarcoma in the anterior mediastinum in a child: report of a case. Surg Today.

[11] Chen HG, Zhang K, Wu WB, Wu YH, Zhang J, Gu LJ, Li XJ (2020). Combining surgery with (125)I brachytherapy for recurrent mediastinal dedifferentiated liposarcoma: a case report and review of literature. World J Clin Cases.

[12] Burt M, Ihde JK, Hajdu SI (1998). Primary sarcomas of the mediastinum: results of therapy. J Thorac Cardiovasc Surg.

